# Recent Advances in the Chemical Biology of *N*-Glycans

**DOI:** 10.3390/molecules26041040

**Published:** 2021-02-16

**Authors:** Asuka Shirakawa, Yoshiyuki Manabe, Koichi Fukase

**Affiliations:** 1 Department of Chemistry, Graduate School of Science, Osaka University, 1-1 Machikaneyama-cho, Toyonaka, Osaka 560-0043, Japan; shirakawaa18@chem.sci.osaka-u.ac.jp; 2 Core for Medicine and Science Collaborative Research and Education, Project Research Center for Fundamental Sciences, Osaka University, 1-1 Machikaneyama-cho, Toyonaka, Osaka 560-0043, Japan

**Keywords:** *N*-glycan, chemical biology, glycan array, NMR, glycoprotein, lectin

## Abstract

Asparagine-linked *N*-glycans on proteins have diverse structures, and their functions vary according to their structures. In recent years, it has become possible to obtain high quantities of *N*-glycans via isolation and chemical/enzymatic/chemoenzymatic synthesis. This has allowed for progress in the elucidation of *N*-glycan functions at the molecular level. Interaction analyses with lectins by glycan arrays or nuclear magnetic resonance (NMR) using various *N*-glycans have revealed the molecular basis for the recognition of complex structures of *N*-glycans. Preparation of proteins modified with homogeneous *N*-glycans revealed the influence of *N*-glycan modifications on protein functions. Furthermore, *N*-glycans have potential applications in drug development. This review discusses recent advances in the chemical biology of *N*-glycans.

## 1. Introduction

**Figure 1:**
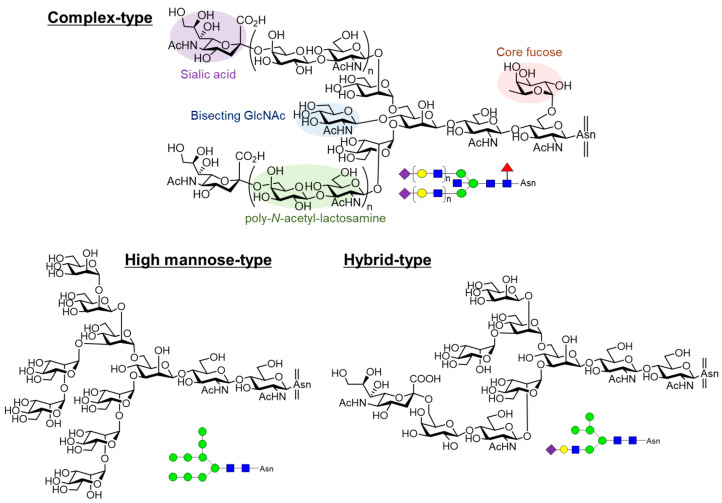
Structures of *N*-glycans. Complex-type *N*-glycans have diverse structures with/without sialic acid, poly-*N*-acetyl-lactosamine, bisecting GlcNAc, core fucose and so on. High-mannose-type *N*-glycan is composed of 14 residues (Glc_3_Man_9_GlcNAc_2_) containing 3 glucoses, 9 mannoses, and 2 GlcNAc. Hybrid-type *N*-glycans have both high-mannose and complex-type structures.

Glycosylation is the most common post-translational modification of proteins. Over 60% of proteins are linked to glycans. Asparagine-linked oligosaccharides (*N*-glycans) have a core pentasaccharide composed of mannose and glucosamine and are classified into three types: high-mannose, hybrid, and complex (Figure 1). In the biosynthesis of *N*-glycan-modified proteins, the high-mannose type *N*-glycan consisting of 14 residues (Glc_3_Man_9_GlcNAc_2_) is first attached to proteins in the endoplasmic reticulum (ER). The initial high-mannose *N*-glycans play an important role in protein folding in the ER. Glycoproteins then migrate to the Golgi apparatus and are subsequently converted into complex-type *N*-glycans. Complex *N*-glycans have diverse structures due to differences in their associated synthesizing enzymes, resulting in different functions for each structure. For example, polylactosamine, consisting of a repeating structure of galactose and glucosamine, is involved in cancer metastasis and immune response [1]. Sialic acids, in contrast, control immunity via recognition by Siglecs expressed in immune cells [2,3]. Core fucose, which is a fucose linked to the glucosamine 6 position at the reducing end, and bisecting glucosamine, which is a glucosamine linked to the branched mannose 4 position, also play various roles and are closely related to many diseases [4]. Hybrid *N*-glycans have both high-mannose and complex-type structures. Thus, *N*-glycans have diverse structures and are involved in a variety of biological phenomena. However, the molecular bases of their modes of action are yet to be fully elucidated.

Chemical synthesis, enzymatic synthesis, and isolation of diverse, pure *N*-glycans have been vigorously investigated for analyzing *N*-glycan functions at the molecular level. Chemical synthesis is an extremely potent approach that allows the de novo construction of glycan structures. Any desired glycan structures can be constructed, including partial and artificial structures. Danishefsky et al. successfully synthesized various *N*-glycans with multiantennary structures [5,6,7,8]. Unverzagt et al. achieved convergent synthesis of complex-type *N*-glycans with bisecting glucosamine and/or core fucose [9,10,11,12]. We have also reported the synthesis of *N*-glycans [13,14]. In addition, Ito et al. [15], Boons et al. [16,17,18], Wang et al. [19], Wong et al. [20,21], and Schmidt et al. [22] have achieved *N*-glycan synthesis. Since the chemical synthesis of *N*-glycans with complex structures is a challenging process that requires multiple steps, the isolation of *N*-glycans from natural resources has also been explored. Kajihara et al. established an efficient method for the isolation of *N*-glycans from egg yolk, which has become a standard method for *N*-glycan preparation [23]. In recent years, the preparation of *N*-glycans using enzymatic reactions has also been extensively investigated [24]. Ito et al. [15], Boons et al. [18,25], Wang et al. [19], and Wong et al. [21] successfully constructed a wide range of *N*-glycan libraries via enzymatic synthesis using isolated or chemically synthesized glycans as substrates. Thus, over the years, the technical basis for a sufficient supply of various *N*-glycans has been established.

**Figure 2:**
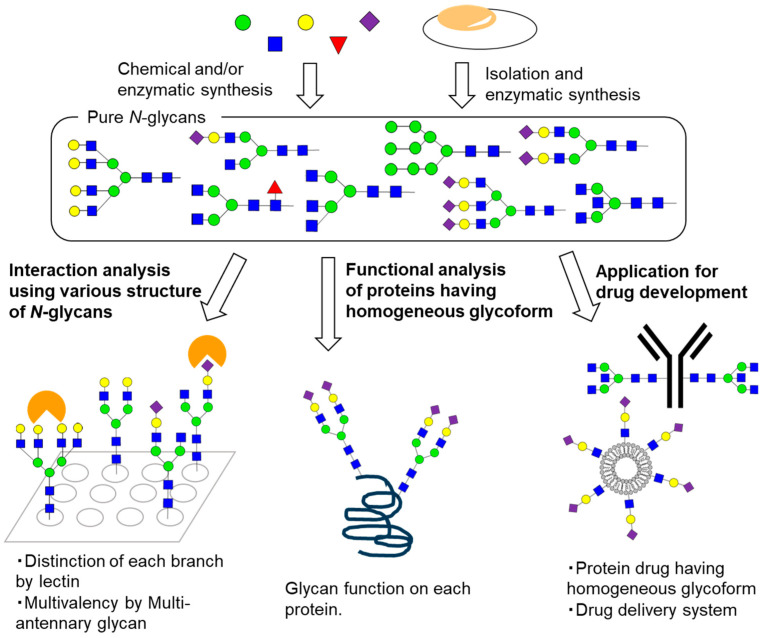
The chemical biology study of homogeneous *N*-glycans. Chemical synthesis, enzymatic synthesis, and isolation of diverse, pure *N*-glycans enable their functional analysis at the molecular level. Interaction analysis using various *N*-glycans revealed the significance of complex *N*-glycan structures—for example, distinction of each branch and multivalent interaction in lectin recognition. Functional analysis using proteins with homogeneous glycoforms clarifies glycan function on each protein. Application of *N*-glycans for drug development is also investigated.

Owing to the increased availability of pure *N*-glycans, their functional elucidation has advanced considerably in recent years (Figure 2). *N*-Glycan functions have mainly been analyzed using molecular biological techniques, including knockout of biosynthetic enzymes. However, it is difficult to determine the precise structure–activity relationship using these methods. Although interaction analysis of lectins using relatively small glycan fragments, such as disaccharides and trisaccharides, has been used to study function, it is not possible to estimate the conformational effect or multivalent interactions of the complex structure of *N*-glycans. Recent interaction analysis of lectins using various *N*-glycans has elucidated the significance of such complex structures. The increased availability of *N*-glycans also allows one to prepare glycoproteins with homogeneous glycoforms, enabling the elucidation of *N*-glycan function on the distinct protein. Furthermore, *N*-glycans have the potential to be used in the development of novel drugs. This review provides an overview of the recent chemical biology study of *N*-glycans.

## 2. Elucidation of the Molecular Basis of *N*-Glycan Recognition by Lectins

Glycans control various biological phenomena through their recognition by lectins. Thus, interaction analysis between *N*-glycans and lectins is essential to elucidate *N*-glycan functions [26]. 

### 2.1. Methods for the Glycan‒Lectin Interaction Analysis

The glycan–lectin interaction analysis methods include analyses using glycan arrays, nuclear magnetic resonance (NMR), isothermal titration calorimetry (ITC) [27], surface plasmon resonance (SPR) [28,29], fluorescent polarization (FP) [30], and X-ray crystallography [31]. ITC gives thermodynamic parameters, whereas SPR provides kinetic parameters. FP realizes a simple and easy assay system. X-ray crystallography provides precise structural information. In this review, we focus on studies using glycan arrays and NMR, which are effective methods for elucidating the interaction between glycans and lectins.

Glycan arrays are used to detect the binding of lectins to immobilized glycans [32,33,34,35]. An advantage of a glycan array is that a large number (dozens and hundreds) of samples can be examined in a high-throughput manner using a small amount of glycans. Interaction analysis using various structures of glycans provides insights into precise structure–activity relationships. Glycan immobilization methods are divided into two categories: noncovalent and covalent [36]. Noncovalent immobilization utilizes hydrophobic interactions, charge interactions, and biotin–streptavidin interactions among others. Covalent immobilization methods typically use coupling of an amino group introduced at the reducing end of a glycan to the plate surface activated with *N*-hydroxysuccinimide [37]. Many other methods, including thiol-maleimide coupling and alkyne-azide click reactions, have been reported. As for the detection, fluorescence is usually used to realize high-throughput analysis. 

**Figure 3:**
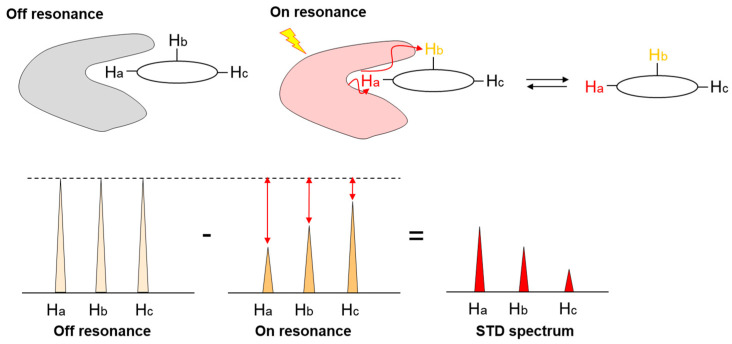
Mechanism of Saturation transfer difference nuclear magnetic resonance (STD-NMR). “Off resonance” experiment gives a reference spectrum. Under “on resonance” conditions, the saturation is transferred from protein to ligand by spin diffusion through intermolecular nuclear Overhauser effects (NOEs). The closer the protons are to the protein, the stronger the STD signals that are observed.

NMR can be used to analyze interactions at the atomic level [38,39]. Saturation transfer difference (STD) NMR [40] is a particularly powerful method for the analysis of glycan–lectin interactions. In this method, saturation transfer from the protein to the ligand is observed as STD signals after the saturation of protein by radio frequency (Figure 3). The closer the protons are to the protein, the stronger the STD signals observed. STD-NMR was originally developed as a method for screening ligands from mixture systems but is now widely used for the analysis of protein–ligand binding modes. This method works when the affinity is not high (K_D_ is 10^−3^ to 10^−8^ M), because STD signals are measured when the protein and ligand are in an equilibrium state of binding and dissociation. Since glycan–lectin interactions are usually weak (K_D_ in mM-μM), STD-NMR is highly effective. NMR is also a powerful tool for the conformational analysis of glycans [41]. Importantly, this method does not require labeled proteins. In addition, a small amount of receptor is necessary (typically micromolar range). However, an excess of the ligands is used (typically the millimolar range), thus, low solubility of the ligand causes a problem. While conformation is an important factor for glycan recognition, the flexibility of glycans makes conformational analysis difficult. In addition to analysis based on coupling constants and the nuclear Overhauser effect (NOE), an analysis using pseudocontact shift (PCS) by paramagnetic metals has recently been developed, and its efficiency has been demonstrated [42,43,44,45,46,47,48].

Examples of the analysis of glycan‒lectin interactions using glycan arrays and NMR are introduced below. 

### 2.2. Analysis of Sugar‒Lectin Interactions Using Glycan Arrays

**Figure 4:**
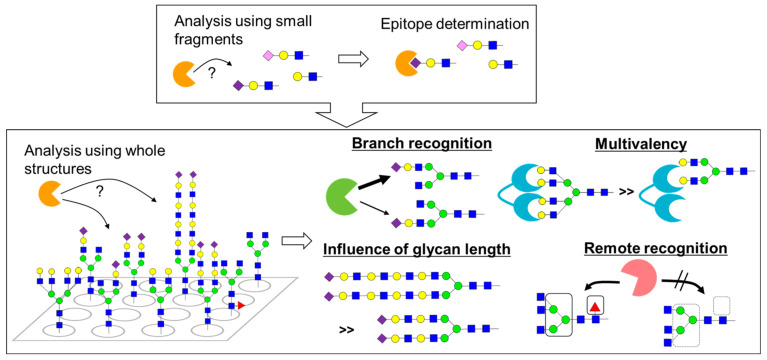
Analysis of glycan‒lectin interaction using glycan arrays. Interaction analysis using small fragments, such as disaccharides and trisaccharides, revealed the epitope required for lectin recognition, whereas interaction analysis using whole structures of *N*-glycans revealed the significance of complexity of *N*-glycan structures; these analyses provided the insights into the differences in the lectin recognition of each branch, the improvement of affinity due to the inclusion of multiple recognition units (multivalent effect), the influence of chain length on affinity, and remote (heterovalent) recognition.

Glycan arrays are excellent tools for the comprehensive analysis of glycan‒lectin interactions. Previous interaction analysis using small fragments, such as disaccharides and trisaccharides, revealed the minimum structure (epitope) required for recognition of individual lectins. Meanwhile, recent advances in the preparation of the whole structure of various *N*-glycans have allowed the full realization of structure–activity relationships, elucidating the significance of complexity of *N*-glycan structures (Figure 4). For example, these advances have provided insights into the differences in the lectin recognition of each branch [49,50,51,52,53,54], the improvement of affinity due to the inclusion of multiple recognition units (multivalent effect) [20,21,55,56,57,58,59], the influence of chain length on affinity [60,61], and remote (heterovalent) recognition [59].

Wang et al. demonstrated the differences of each *N*-glycan branch in lectin recognition by comprehensive interaction analysis of various *N*-glycans with several lectins using a glycan array [53,54]. Plant-derived *Sambucus nigra* lectin (SNA), which recognizes sialic acid, recognized the sialic acid on the α1,3-branched chain more strongly than the sialic acid on the α1,6-branched chain. Meanwhile, plant-derived *Maackia amurensis* lectin (MAL-I) and virus-derived lectin hemagglutinin (HA) strongly bind to sialic acid on the α1,6-branched chain. MAL-I also interacts with terminal galactose; in this case, MAL-I strongly recognizes galactose on the α1,3-branched chain, suggesting that MAL-I has two distinct glycan recognition domains. In addition, *Erythrina cristagalli* lectin (ECL), which recognizes the lactosamine structure, has a higher affinity to lactosamine on the α1,3-branched chain than on the α1,6-branched chain. *Phaseolus vulgaris* erythroagglutinin (PHA-E) prefers terminal galactose on the α1,6-branched chain and terminal glucosamine on the α1,3-branched chain, whereas wheat germ agglutinin (WGA) strongly interacts with glucosamine on the α1,3-branched chain. 

Branch selective binding of C-type lectins and monoclonal antibodies was also revealed by using glycan array including *N*-glycan positional isomers prepared by chemo-enzymatic method [50]. DC-SIGN, C-type lectin recognizing glycan on bacteria and viruses, showed strong binding to hybrid- and complex-type glycans and *N*-glycans presenting Lex epitopes. DC-SIGN showed preferential binding to the biantennary glycans with terminal galactose or *N*-acetylgalactosamine on the α1,6-branched chain, whereas DC-SIGNR showed the opposite binding behavior. L-SECtin showed the preference to GlcNAc1,2-Man residues on the 3-arm of the complex and hybrid *N*-glycans.

*N*-glycans have symmetric structures on the nonreducing end side, and these studies indicate that glycan structures on each branched chain have distinct functions.

The structural redundancy of *N*-glycans plays an important role in enhancing their affinity to lectins because of their multivalency. Interaction analysis of Siglec-1, -2, -9, and -10 with sialic acid-containing *N*-glycans using a glycan array showed a higher affinity for four-branching *N*-glycans than for two-branching *N*-glycans [59]. Multivalent effects were also confirmed in ECL, which recognizes the lactosamine structure, and *Ricinus communis* agglutinin (RCA120), which recognizes terminal galactose [58]. Similarly, interactions with galectin were also enhanced as the number of recognition units increased [62].

The structure at the remote positions of the lectin recognition unit can affect its interaction. *Lens culinaris* agglutinin (LCA), which binds to core fucose, only recognizes core fucosylated biantennary and triantennary *N*-glycans with particular branching patterns, but did not recognize triantennary *N*-glycans with other branching patterns or tetraantennary *N*-glycans [59]. These results indicate that the branching structure away from the core fucose affected recognition by LCA, although its recognition site is core fucose. On the other hand, the affinity between HA from H3N3 and sialic acids at the nonreducing end was increased by chain elongation; the insertion of a polylactosamine repeating structure enhanced the affinity [60]. These studies revealed that both the epitope and the whole glycan structure are important for the recognition of *N*-glycans.

Glycan arrays, comprising *N*-glycans along with glycolipids and *O*-glycans, have been used to investigate the host–pathogen interactions in diagnostic and therapeutic applications [63,64,65,66,67,68,69]. For example, the inhibition of human anti-N9 antibodies to influenza neuraminidases was analyzed by glycan array [70]. The binding study of the H3N2 influenza viruses using glycan microarrays demonstrated the changes in virus hemagglutinin that affect the receptor binding properties of the viruses [71].

Glycan arrays can also be used to explore artificial glycoligands as new drug candidates that target lectins [72,73,74]. High-affinity ligands for Siglecs or several C-type lectins, which are involved in immune regulation, are expected to be lead compounds for drug development. However, glycan–lectin interactions are usually weak, which is a major issue in the utilization of glycans as bioactive molecules. Thus, the synthesis of glycans and derivatization of artificial molecules, followed by high-throughput screening using glycan arrays, is expected to be a powerful approach to address this limitation.

### 2.3. Analysis Using NMR

NMR analysis can provide insights into glycan–lectin interactions at the atomic level. STD-NMR can be used for high-resolution epitope mapping. Similar to the results obtained from glycan arrays, NMR analysis also reveals that not only epitopes but the whole structure of *N*-glycans plays an important role in glycan–lectin interactions. The conformational analysis of *N*-glycans using NMR is a powerful approach that provides a rational explanation for the molecular basis of the recognition of complexity of *N*-glycan structures.

STD-NMR allows for a detailed analysis of glycan–lectin interactions. Many researchers have analyzed the interactions between sialic acid containing *N*-glycans and Siglecs [75,76,77,78,79]. Silipo et al. analyzed the interaction between Siglec-2 and sialyl *N*-glycans using STD-NMR and molecular dynamics (MD) simulations [78]. The Siglec-2 epitope was clearly shown by STD-NMR, and the conformation of sialyl *N*-glycans was predicted by NMR analysis and MD simulations. When biantennary sialyl *N*-glycans were recognized, Siglec-2 only interacted with the sialyl disaccharide at the nonreducing end, and the other part was expected to protrude from the protein surface. These results suggest that multiantennary *N*-glycans with multiple sialic acids can interact with several Siglec-2 and induce the formation of Siglec-2 oligomers on B cells.

STD-NMR analysis using the whole structure of *N*-glycans has demonstrated that lectins not only recognize small units, such as disaccharides and trisaccharides, but also interact with *N*-glycans in a more complex manner. In *N*-glycan recognition by *Pisum sativum* agglutinin (PSA), a mannose-recognition lectin, core fucose was shown to alter its binding mode [80]. When biantennary *N*-glycans without core fucose were used for the interaction analysis with PSA, the mannose on each branch gave comparable STD signals. While for the core fucose containing *N*-glycans, the STD signals of mannose on the α1,6-branched chain were weakened, and instead, an interaction with the methyl group of the core fucose was observed. STD-NMR using a fluorine derivative (2D STD-TOCSYreF) indicated that the mannose on the α1,3-branched chain was more strongly recognized by PSA than the mannose on the α1,6-branched chain [81]. In addition, dectin-1, which recognizes fungal β-glucan, was found to recognize core fucose on immunoglobulin (IgG) [82]. STD-NMR analysis indicated that dectin-1 interacted not only with core fucose but also with an Fmoc group attached to the amino group of asparagine introduced at the reducing end. These results suggest that dectin-1 recognizes amino acids with aromatic side chains, such as phenylalanine and tyrosine, together with core fucose. On the other hand, STD-NMR is also effective for the analysis of substrate recognition by glycosyltransferases. The STD-NMR analysis of FUT8, a fucosyltransferase that builds core fucose structure, revealed the precise interaction between FUT8 and *N*-glycan [83]. FUT8 recognizes not only glucosamine at the reducing end (reaction point) but also the whole glycan structure. In particular, FUT8 strongly interacted with the α1,3-branched chain at the nonreducing end.

Advanced STD-NMR methods have been developed. Saturation transfer double difference (STDD)-NMR is useful for the direct observation of ligands binding on the surfaces of living cells [84]. Clean-STD can avoid accidental saturation to give improved detection of ligand–protein interactions at low concentration of protein [85]. Second dimension STD-NMR, i.e., STD-TOCSY, STD-HSQC, STD-NOESY, can overcome the problems of proton overlapping typical of glycan NMR analysis [86].

**Figure 5:**
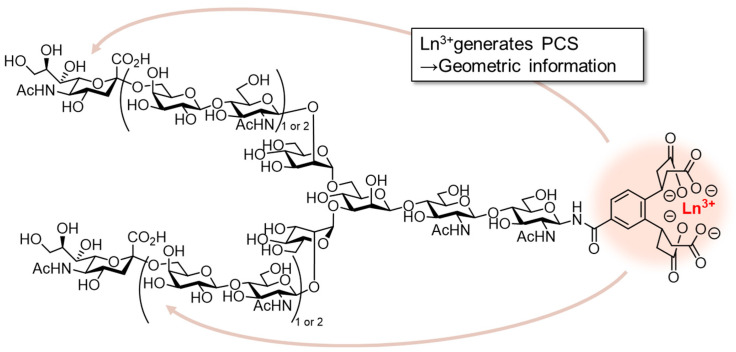
Conformation analysis of *N*-glycan using pseudocontact shift (PCS). Chelation with paramagnetic metals can induce PCS to give geometric information of *N*-glycan.

**Figure 6:**
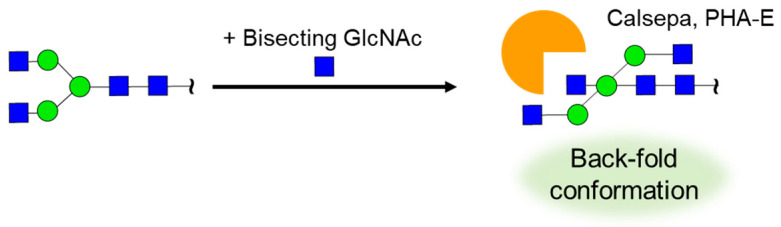
Recognition of bisecting GlcNAc containing *N*-glycan by Calsepa and *Phaseolus vulgaris* erythroagglutinin (PHA-E). Attachment of bisecting GlcNAc enhances back-fold conformation, which is recognized by Calsepa and PHA-E.

Conformation analysis of glycans using NMR provides important insights into complex glycan–lectin interactions. The *N*-glycan conformation can be predicted by combining PCS-based NMR analysis and MD simulations (Figure 5) [42,43,44,45,46,47,48]. Kato et al. analyzed the conformation of high-mannose glycans by PCS-based NMR analysis using ^13^C-labeled compounds and Tm^3+^ as a paramagnetic metal ion tag [38]. They elucidated the conformational change caused by mannose trimming during the *N*-glycan biosynthetic process. Unverzagt and Barbero et al. distinguished each branch of tetraantennary *N*-glycan based on the PCS method and analyzed the differences in the recognition of each branch by lectins [44]. *Datura stramonium* seed lectin (DSL), which recognizes the lactosamine structure, interacts more strongly with the lactosamine on the α1,6-branched chain than with that on the α1,3-branched chain. On the other hand, no differences in the strengths of STD signals of each branch were observed with *Ricinus communis* agglutinin (RCA120), which recognizes terminal galactose, indicating that RCA120 recognizes all branches without distinction. In a similar analysis between sialic acid containing biantennary *N*-glycans and HA, STD signals from both sialic acids were observed, suggesting the contribution of two sialic acids in a multivalent effect [45]. Furthermore, interesting results have been reported showing that the *N*-glycan conformation directly affects lectin recognition (Figure 6) [87]. *N*-Glycans have three back-fold conformations and two extended conformations, in which the α1,6-branched chain is folded toward the reducing end or extended, respectively. The addition of core fucose or bisecting glucosamine significantly changes their conformational equilibria and reduces the number of major conformations from five to four and five to two, respectively [88,89]. Crystal structure analysis and transferred NOE (TrNOE) analysis revealed that *Calystegia sepium*-derived calsepa and *Phaseolus vulgaris*-derived phytohemagglutinin (PHA-E), which recognize bisecting glucosamine containing *N*-glycans, recognize *N*-glycans in the back-fold conformation induced by bisecting glucosamine addition.

## 3. Functional Analysis of *N*-glycans on Glycoproteins

Analysis of *N*-glycan functions on glycoproteins needs to be considered with proteins. In recent years, improvements in the techniques for the synthesis of peptides and proteins, as well as glycans, have enabled the preparation of glycoproteins with homogeneous glycans [90,91,92,93,94]. *N*-Glycans on glycoproteins can be modified by Endo-β-*N*-acetylglucosaminidases (ENGases) [95]. Synthesized glycoproteins with homogeneous glycan structures have helped elucidate precise glycan functions. 

**Figure 7:**
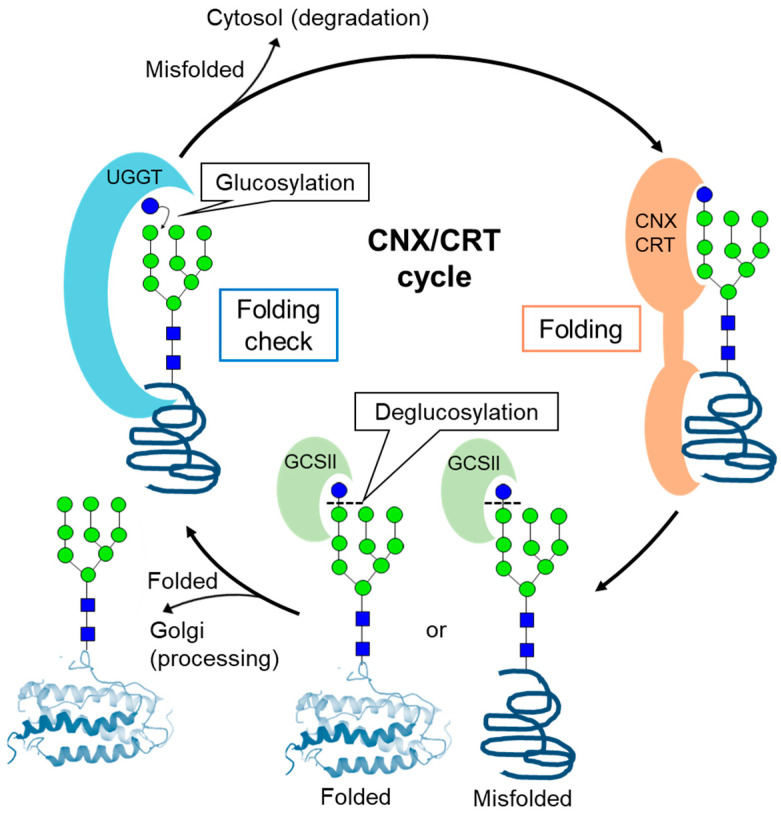
Protein quality control utilizing high-mannose-type *N*-glycan as a tag. UDP-glucose:glycoprotein glucosyltransferase (UGGT) complex distinguishes misfolded glycoproteins to transfer glucose to the nonreducing end of the high-mannose glycan. This monoglycosylation serves as a marker for misfolded glycoproteins and the chaperone proteins calnexin/calreticulin (CNT/CRT) promotes folding.

A series of synthetic studies of glycoproteins and glycoprotein mimics by Ito and Kajihara et al. revealed the precise function of *N*-glycans in a quality-control mechanism for glycoproteins in the endoplasmic reticulum (ER). (Figure 7) [96,97]. ER has a quality control system that promotes the correct folding of ribosome-produced proteins. In the case of *N*-glycosylated proteins, high-mannose *N*-glycans work as tags for protein folding. A common dolichol-linked oligosaccharide precursor containing terminal glucose trisaccharide is first synthesized in the ER and is transferred to proteins by the oligosaccharyltransferase (OST). The folding process then starts. The first glycosidase (GCSI) cleaves the terminal glucose and the second glycosidase (CGSII) further cleaves glucose residues to afford monoglucosylated or nonglucosylated glycoproteins. The folded nonlucosylated glycoproteins are then transferred to the glycan modification process. The UDP-glucose:glycoprotein glucosyltransferase (UGGT) complex distinguishes misfolded glycoproteins and transfers glucose to the nonreducing end of the high-mannose glycan. This monoglucosylation serves as a marker for misfolded glycoproteins and the chaperone proteins calnexin/calreticulin (CNT/CRT) promotes folding. CGSII then cleaves glucose residue to transfer the glycoproteins for the glycan modification process. 

The defects in this process cause congenital disorders of glycosylation (CDGs), which are severe genetic diseases [98]. CDG is classified into Type I and Type II. In Type I, the enzymes are mutated in synthesis and transfer a common dolichol-linked oligosaccharide precursor and enzyme substrates. Type II defects the modification process of *N*-glycans in the ER and Golgi. Lack of GCS1 causes CDG-IIb. Unfolded proteins lead to ER stress and cause CDGs [99]. 

Ito et al. introduced methotrexate (MTX) at the reducing end of high-mannose *N*-glycans and prepared a complex with dihydrofolate reductase (DHFR), which recognizes MTX [100,101]. Such glycoprotein mimics were used to analyze the interaction with UGGT. They also investigated various aglycone structures as substrates of UGGT [102,103,104]. In addition, chemically synthesized glycoproteins were used for the analysis of substrate recognition by UGGT. UGGT showed higher enzymatic activity against high-mannose *N*-glycans on misfolded interleukin-8 (IL-8) than against those on the folded one [105]. Furthermore, they synthesized several glycoproteins and isotope-labeled glycopeptides and revealed that UGGT recognizes hydrophobic patches on misfolded proteins [106,107]. As shown above, they elucidated the molecular basis of the quality-control mechanism based on high-mannose *N*-glycans using glycoprotein mimics and chemically synthesized glycoproteins.

Maintaining the appropriate folding is also critical for in the degradation process. Mutations in human *N*-glycanase 1 (NGLY1) cause the congenital disorder of deglycosylation (CDDG). Suzuki revealed that *N*-GlcNAc proteins are accumulated by the action of Endo-β-*N*-acetylglucosaminidase (ENGase) in Ngly1-defective cells [108,109]. During ER-associated degradation, *N*-GlcNAc proteins form aggregates that seem to be toxic. Suzuki also revealed that lethality of Ngly1-KO mice is partially rescued by the additional deletion of the Engase gene, suggesting that ENGase inhibitors are targets for CDDG [110,111].

In recent years, the influence of *N*-glycan modifications on the bioactivity of proteins has been gradually elucidated using synthetic glycoproteins [112,113,114,115,116,117]. Hematopoietic hormone erythropoietin (EPO), which is used to treat renal anemia, has three *N*-glycan-modification sites. EPO with various glycoforms is used as a drug. Several groups have reported the synthesis of EPO with homogeneous glycoforms, and the effect of *N*-glycans on their biological activities has been investigated [112,115,116,118,119,120]. In addition, various neoglycoprotein analogues of EPO have been reported [121,122,123,124]. Kajihara et al. synthesized five types of EPO, which is introduced sialic acid containing *N*-glycans into three *N*-glycosylation sites with different patterns, and showed the relationship between glycosylation sites and hematopoietic activities [115]. Increasing the number of sialic acids containing *N*-glycans on EPO improved the stability in blood, leading to an improvement in hematopoietic activity. Moreover, the metabolic stability of EPO was highly correlated with hydrophobicity, suggesting that glycan modifications enhance the in vivo stability by covering hydrophobic sites on the protein surface. Kajihara et al. also synthesized two types of interferon-β (IFN-β) with sialic acid-containing and noncontaining (asialo) *N*-glycans, and their activities were evaluated [117]. IFN-β modified with sialic acid-containing *N*-glycans exhibited higher activity than that modified with asialo *N*-glycans, suggesting that sialic acid extended the in vivo half-life of IFN-β. Thus, *N*-glycans are closely related to the stability of glycoproteins in vivo. Indeed, Tanaka et al. demonstrated the effect of *N*-glycans on protein metabolic stability by positron emission tomography (PET) imaging using glycodendrimers as pseudoglycoproteins [125,126]. On the other hand, *N*-glycosylation can also affect binding affinity to a receptor. Okamoto et al. synthesized two types of chemokine CCL1 with and without *N*-glycan [113], in which *N*-glycosylation reduced the activity of CCL1, suggesting that CCL1 biological activity can be regulated by *N*-glycan modification. Thus, it should be noted that the role of *N*-glycan modifications can be different between proteins. We reported that dectin-1 specifically recognized core fucosylated IgG and did not interact with other core fucosylated proteins, suggesting that core fucose on IgG has specific physiological functions [82]. The role of *N*-glycans on distinct proteins is an important topic for future work.

## 4. Use of *N*-glycans for Drug Development

The increased supply of *N*-glycans has led to an increase in the use of *N*-glycans for drug development [127]. Because *N*-glycans are endogenous molecules, they are unlikely to be toxic or immunogenic and, thus, are expected to have high safety profiles.

### 4.1. Next-Generation Protein/Peptide Drugs Modified with Homogeneous N-Glycans

Controlling the glycan structure is an important issue in the preparation of glycoprotein and glycopeptide drugs. Biopharmaceuticals, including antibodies, are common pharmaceuticals. Although many proteins utilized in biopharmaceuticals are glycoproteins, their actual glycan structures are often neglected or ignored. However, the significance of the role of glycans on the function of glycoproteins has recently been illuminated, and the importance of the glycan structure has been highlighted. The preparation of glycoproteins with homogeneous glycans is also important from the viewpoint of quality control.

**Figure 8:**
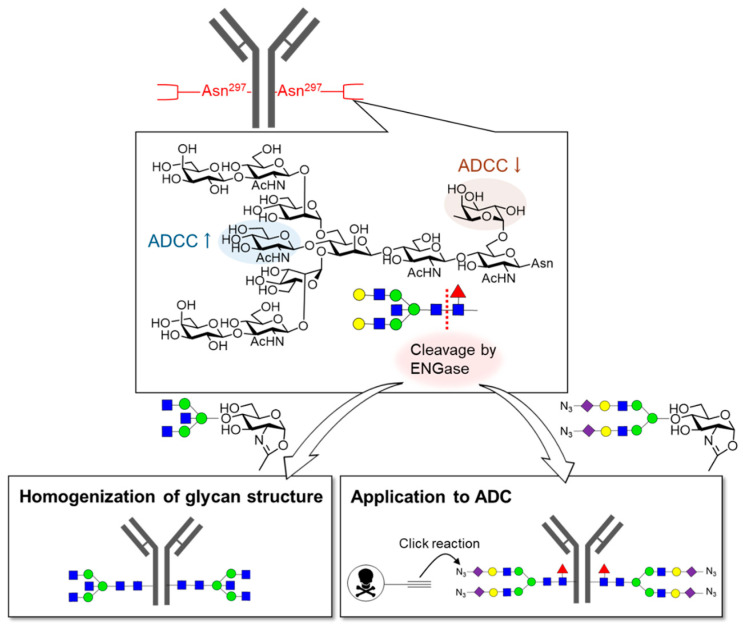
Glycan editing of immunoglobulin (IgG) antibodies. *N*-Glycans at Asn297 of IgG affect their activity. Core fucose reduces the antibody-dependent cellular cytotoxicity (ADCC) activity, whereas bisecting GlcNAc enhances the ADCC activity. *N*-Glycan editing using Endo-β-*N*-acetylglucosaminidase (ENGase) can give IgG as a homogeneous glycoform or can be applied for the preparation of Antibody–drug conjugates (ADCs).

IgG antibodies have *N*-glycans at Asn297 in the Fc region of the heavy chain, and their structures affect activity, dynamics, and safety (Figure 8) [128,129]. The importance of core fucose on these *N*-glycans is well known. The removal of the core fucose from IgG antibodies dramatically enhances antibody-dependent cellular cytotoxicity (ADCC) activity [130,131,132,133]. Mogamulizumab, the antibody without core fucose, is actually in current use. Bisecting glucosamine and terminal galactose have been reported to affect ADCC and complement-dependent cytotoxicity (CDC) activities [134,135,136]. Therefore, modifications of *N*-glycans on IgG antibodies have been extensively investigated. ENGase provides a powerful tool [137]. *N*-Glycans on antibodies can be trimmed, and other *N*-glycans can be introduced by ENGase. Antibody–drug conjugates (ADCs) have also been prepared using this method [138,139,140] in which *N*-glycans were changed into a structure with a tag for subsequent reactions, and small molecular drugs were introduced via bio-orthogonal reactions [141,142,143,144,145,146]. This approach allows for the introduction of drugs into the Fc region without affecting antigen recognition. Furthermore, the *N*-glycan structure can be made homogeneous. Wong et al. introduced *N*-glycans with 3-position fluorinated sialic acids into antibodies [147]. Because this fluorinated *N*-glycan was not degraded by sialidase and modification with sialic acid containing *N*-glycan can enhance the metabolic stability of proteins, this antibody is expected to show a significant improvement in pharmacokinetics.

*N*-Glycans play important roles not only in antibodies, but also in many other glycoproteins. As described above, the structure–activity relationship study of *N*-glycans on EPO demonstrates the importance of the *N*-glycan structure on the bioavailability and bioactivity of proteins [115]. Hossain and Wade et al. reported that the physical properties of insulin can be improved by adding *N*-glycan to insulin, which originally has no glycans [148]. Introduction of sialic acid containing *N*-glycans to insulin successfully inhibited problematic fibril formation. In addition, *N*-glycan-modified insulin bound to its receptor with almost the same affinity as the natural form, and further improvements in its metabolic stability were observed. Currently, PEGylation has been generally used to enhance the bioavailability of proteins; however, PEG is not without adverse effects. Considering that *N*-glycans are endogenous glycans and are expected to be extremely safe, “*N*-glycan modification” has the potential to become a common strategy for improving the protein/peptide bioactivity.

The structure of *N*-glycans is also important for vaccine development. Viruses use host biosynthetic systems to synthesize proteins. Consequently, viral proteins are subjected to glycan modification. Therefore, glycoproteins and glycopeptides are candidate antigens for vaccine development, and their glycan structures influence their functions. HIV vaccine candidates containing *N*-glycans have been designed and synthesized [149,150,151,152,153]. Wang et al. reported that the glycan structure on the antigen was critical for the neutralization activity of antibodies, clearly demonstrating the importance of the glycan structure in vaccine design [150]. In addition, Wang showed the importance of glycan structures in the development of influenza HA-based vaccines [154,155]. For the development of vaccines against COVID-19, the spike protein is a promising antigen candidate. This protein is heavily glycosylated [156], but *N*-glycan modifications of spike proteins have been reported to reduce their antigenicity [157]. However, *N*-glycan-modified antigens may induce antibodies against endogenous *N*-glycans, which should be carefully examined. Overall, glycans are likely to be important for developing highly efficient and safe vaccines.

### 4.2. Drug Delivery Systems (DDSs) Using N-Glycans

*N*-Glycans interact with various biomolecules, including many lectins, and thus show distinct dynamics in vivo. Therefore, DDSs using *N*-glycans have been investigated [126,158,159]. Because glycan–lectin interactions are weak, multivalent materials, including polymers, dendrimers, and liposomes, are usually utilized to enhance their interactions [160].

**Figure 9:**
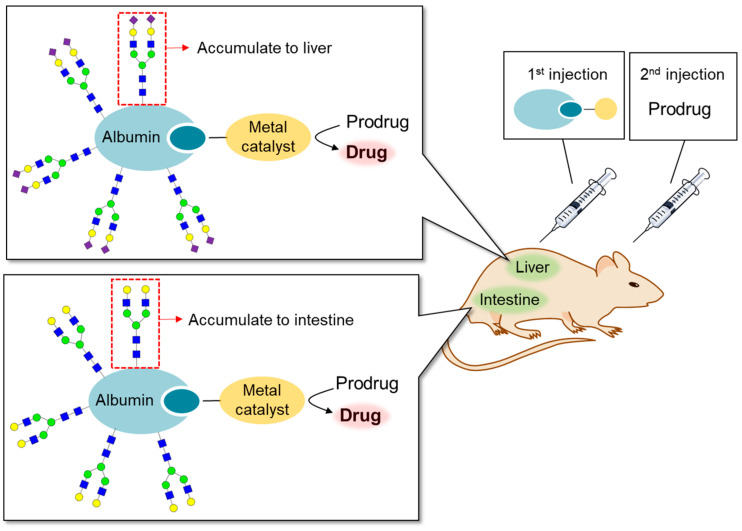
In vivo reaction using artificial glycosylated albumin metalloenzymes. Specific *N*-glycan conjugated albumin is specifically uptaken into the specific organs. Thus, the albumins conjugated with *N*-glycans were used as carriers of metal catalysts to realize chemical reactions for the activation of prodrug at the desired organ.

We synthesized dendrimers of sialic acid containing *N*-glycans and evaluated their dynamics in vivo using PET imaging [125]. We revealed that the structure of *N*-glycans affected the uptake of dendrimers into specific organs. In addition, Tanaka et al. developed an *N*-glycan-based DDS using albumin as a multivalent scaffold (Figure 9). The albumins modified with *N*-glycans were used as carriers of metal catalysts to realize chemical reactions at the desired organ in vivo [161,162,163]. It should be noted that they achieved metal-catalyzed reactions in vivo by utilizing the hydrophobic pocket of albumin.

Siglecs, which recognize sialic acid, are expressed on immune cells and are involved in immune regulation [2,3]. Immune cells can be targeted by utilizing sialyl glycan–Siglec interactions [164,165,166,167,168,169,170]. Paulson et al. developed high-affinity Siglec ligands by the derivatization of sialic acid. They synthesized *N*-glycans containing these artificial structures, which exhibited a high affinity for Siglec-2 [168]. They achieved B cell targeting using liposomes displaying this *N*-glycan. Utilization of different sialyl glycans enables the targeting of various immune cells. In addition to Siglecs, DDSs targeting galectins, which recognize galactose, have also been investigated [171,172,173].

## 5. Future Perspectives

Glycans exist as polysaccharides in nature and are involved in multivalent interactions for pattern recognition. Conformational control via the formation of polysaccharides also plays an important role in glycan functions. In addition, many glycans function only when they are linked to proteins or lipids. Such emergent glycan functions can only be revealed by analysis using the whole glycan structure or glycoconjugates. As described herein, the increased availability of various *N*-glycans has led to the elucidation of the significance of complex *N*-glycan structures. The influence of *N*-glycan modification on some protein functions was also discussed. On the other hand, the molecular basis of glycan functions on membrane proteins remains to be elucidated, although glycans are attached to almost all membrane proteins and have diverse functions. Recent advances in the engineering of cell-surface glycans [174] are expected to provide a powerful approach to tackle this challenging issue. Bertozzi et al. developed metabolic labeling of cell surface glycan by incorporating unnatural sugar analogs having the reaction tag followed by the bio-orthogonal reaction [175]. This method enables the installation of chemical functionality, i.e., fluorescent group, to glycans. In addition to glycan function analysis, the therapeutic application of metabolic glycan labeling is being vigorously investigated [176]. Glycan engineering by chemical [177,178] and chemoenzymatic [179,180,181,182,183] methods has also been investigated. In addition, de novo glycans on cell surfaces have also been reported, such as the direct introduction of defined glycan structures into plasma membranes by lipid insertion, liposomal fusion, and tag technology [184,185,186,187,188,189]. Such glycan editing technique enables glycan functions to be explored on membrane proteins on living cell surfaces.

A major feature of glycans is their heterogeneity. Glycans attached to the same site on the same protein can have diverse structures. In addition, many proteins have multiple glycosylation sites to which various glycans can be added. Although studies using pure *N*-glycans have revealed the functions of individual *N*-glycans, little is known about their function in combination with each other. Kurbangalieva and Tanaka et al. prepared albumins labeled with several *N*-glycans and observed their dynamics in vivo. Interestingly, their dynamics were altered depending on the *N*-glycosylation pattern [190]. These results suggest that the simultaneous interaction of multiple *N*-glycans may result in the expression of functions different from those of individual *N*-glycans. Little is known about whether the interactions of glycans with multiple lectins work collaboratively or competitively. A bottom-up approach to the construction of controlled glycoforms is expected to be a powerful strategy to address this difficult issue.

Glycans are considered to be the third most important life chain and have attracted increasing attention in recent years. However, unlike nucleic acids and proteins, their functional analysis and regulation have been delayed due to the lack of simple preparation methods. Recent advances in the preparation of *N*-glycans are expected to accelerate functional studies.

## Sample Availability

Samples of the compounds are not available from the authors.

## References

[1] Dennis J.W., Granovsky M., Warren C.E. (1999). Glycoprotein glycosylation and cancer progression. Biochim. Biophys. Acta. Gen. Subj..

[2] Crocker P.R., Paulson J.C., Varki A. (2007). Siglecs and their roles in the immune system. Nat. Rev. Immunol..

[3] Macauley M.S., Crocker P.R., Paulson J.C. (2014). Siglec-mediated regulation of immune cell function in disease. Nat. Rev. Immunol..

[4] Takahashi M., Kuroki Y., Ohtsubo K., Taniguchi N. (2009). Core fucose and bisecting GlcNAc, the direct modifiers of the N-glycan core: Their functions and target proteins. Carbohydr. Res..

[5] Wu B., Hua Z., Warren J.D., Ranganathan K., Wan Q., Chen G., Tan Z., Chen J., Endo A., Danishefsky S.J. (2006). Synthesis of the fucosylated biantennary N-glycan of erythropoietin. Tetrahedron Lett..

[6] Wang P., Zhu J., Yuan Y., Danishefsky S.J. (2009). Total Synthesis of the 2,6-Sialylated Immunoglobulin G Glycopeptide Fragment in Homogeneous Form. J. Am. Chem. Soc..

[7] Walczak M.A., Danishefsky S.J. (2012). Solving the Convergence Problem in the Synthesis of Triantennary N-Glycan Relevant to Prostate-Specific Membrane Antigen (PSMA). J. Am. Chem. Soc..

[8] Walczak M.A., Hayashida J., Danishefsky S.J. (2013). Building Biologics by Chemical Synthesis: Practical Preparation of Di- and Triantennary N-Linked Glycoconjugates. J. Am. Chem. Soc..

[9] Schuberth R., Unverzagt C. (2005). Synthesis of a N-glycan nonasaccharide of the bisecting type with additional core-fucose. Tetrahedron Lett..

[10] Eller S., Schuberth R., Gundel G., Seifert J., Unverzagt C. (2007). Synthesis of Pentaantennary N-Glycans with Bisecting GlcNAc and Core Fucose. Angew. Chem. Int. Ed..

[11] Mönnich M., Eller S., Karagiannis T., Perkams L., Luber T., Ott D., Niemietz M., Hoffman J., Walcher J., Berger L. (2016). Highly Efficient Synthesis of Multiantennary Bisected N-glycans Based on Imidates. Angew. Chem. Int. Ed..

[12] Luber T., Niemietz M., Karagiannis T., Mönnich M., Ott D., Perkams L., Walcher J., Berger L., Pischl M., Weishaupt M. (2018). A Single Route to Mammalian N-Glycans Substituted with Core Fucose and Bisecting GlcNAc. Angew. Chem. Int. Ed..

[13] Nagasaki M., Manabe Y., Minamoto N., Tanaka K., Silipo A., Molinaro A., Fukase K. (2016). Chemical Synthesis of a Complex-Type N-Glycan Containing a Core Fucose. J. Org. Chem..

[14] Manabe Y., Shomura H., Minamoto N., Nagasaki M., Takakura Y., Tanaka K., Silipo A., Molinaro A., Fukase K. (2018). Convergent Synthesis of a Bisecting N-Acetylglucosamine (GlcNAc)-Containing N-Glycan. Chem. Asian J..

[15] Koizumi A., Matsuo I., Takatani M., Seko A., Hachisu M., Takeda Y., Ito Y. (2013). Top-Down Chemoenzymatic Approach to a High-Mannose-Type Glycan Library: Synthesis of a Common Precursor and Its Enzymatic Trimming. Angew. Chem. Int. Ed..

[16] Wang Z., Chinoy Z.S., Ambre S.G., Peng W., McBride R., de Vries R.P., Glushka J., Paulson J.C., Boons G.-J. (2013). A General Strategy for the Chemoenzymatic Synthesis of Asymmetrically Branched N-Glycans. Science.

[17] Li T., Huang M., Liu L., Wang S., Moremen K.W., Boons G.-J. (2016). Divergent Chemoenzymatic Synthesis of Asymmetrical-Core-Fucosylated and Core-Unmodified N-Glycans. Chem. Eur. J..

[18] Gagarinov I.A., Li T., Toraño J.S., Caval T., Srivastava A.D., Kruijtzer J.A.W., Heck A.J.R., Boons G.-J. (2017). Chemoenzymatic Approach for the Preparation of Asymmetric Bi-, Tri-, and Tetra-Antennary N-Glycans from a Common Precursor. J. Am. Chem. Soc..

[19] Li L., Liu Y., Ma C., Qu J., Calderon A.D., Wu B., Wei N., Wang X., Guo Y., Xiao Z. (2015). Efficient chemoenzymatic synthesis of an N-glycan isomer library. Chem. Sci..

[20] Shivatare S.S., Chang S.-H., Tsai T.-I., Ren C.-T., Chuang H.-Y., Hsu L., Lin C.-W., Li S.-T., Wu C.-Y., Wong C.-H. (2013). Efficient Convergent Synthesis of Bi-, Tri-, and Tetra-antennary Complex Type N-Glycans and Their HIV-1 Antigenicity. J. Am. Chem. Soc..

[21] Shivatare S.S., Chang S.-H., Tsai T.-I., Tseng S.Y., Shivatare V.S., Lin Y.-S., Cheng Y.-Y., Ren C.-T., Lee C.-C.D., Pawar S. (2016). Modular synthesis of N-glycans and arrays for the hetero-ligand binding analysis of HIV antibodies. Nat. Chem..

[22] Chiesa M.V., Schmidt R.R. (2000). Synthesis of an Asparagine-Linked Heptasaccharide—Basic Structure of N-Glycans. Eur. J. Org. Chem..

[23] Kajihara Y., Suzuki Y., Yamamoto N., Sasaki K., Sakakibara T., Juneja L.R. (2004). Prompt Chemoenzymatic Synthesis of Diverse Complex-Type Oligosaccharides and Its Application to the Solid-Phase Synthesis of a Glycopeptide with Asn-Linked Sialyl-undeca- and Asialo-nonasaccharides. Chem. Eur. J..

[24] Chao Q., Ding Y., Chen Z.-H., Xiang M.-H., Wang N., Gao X.-D. (2020). Recent Progress in Chemo-Enzymatic Methods for the Synthesis of N-Glycans. Front. Chem..

[25] Li T., Liu L., Wei N., Yang J.-Y., Chapla D.G., Moremen K.W., Boons G.-J. (2019). An automated platform for the enzyme-mediated assembly of complex oligosaccharides. Nat. Chem..

[26] Paulson J.C., Blixt O., Collins B.E. (2006). Sweet spots in functional glycomics. Nat. Chem. Biol..

[27] Dam T.K., Talaga M.L., Fan N., Brewer C.F., Feig A.L. (2016). Chapter Four—Measuring Multivalent Binding Interactions by Isothermal Titration Calorimetry. Methods Enzymol.

[28] Duverger E., Frison N., Roche A.-C., Monsigny M. (2003). Carbohydrate-lectin interactions assessed by surface plasmon resonance. Biochimie.

[29] Safina G. (2012). Application of surface plasmon resonance for the detection of carbohydrates, glycoconjugates, and measurement of the carbohydrate-specific interactions: A comparison with conventional analytical techniques. A critical review. Anal. Chim. Acta.

[30] Kakehi K., Oda Y., Kinoshita M. (2001). Fluorescence Polarization: Analysis of Carbohydrate–Protein Interaction. Anal. Biochem..

[31] Nagae M., Yamaguchi Y. (2012). Function and 3D Structure of the N-Glycans on Glycoproteins. Int. J. Mol. Sci..

[32] Feizi T., Fazio F., Chai W., Wong C.-H. (2003). Carbohydrate microarrays—A new set of technologies at the frontiers of glycomics. Curr. Opin. Struct. Biol..

[33] Liang P.-H., Wu C.-Y., Greenberg W.A., Wong C.-H. (2008). Glycan arrays: Biological and medical applications. Curr. Opin. Chem. Biol..

[34] Rillahan C.D., Paulson J.C. (2011). Glycan Microarrays for Decoding the Glycome. Annu. Rev. Biochem.

[35] (2007). Analysis of Glycans, Polysaccharide Functional Properties & Biochemistry of Glycoconjugate Glycans, Carbohydrate-Mediated Interactions: Comprehensive Glycoscience: From Chemistry to Systems Biology.

[36] Wang D., Liu S., Trummer B.J., Deng C., Wang A. (2002). Carbohydrate microarrays for the recognition of cross-reactive molecular markers of microbes and host cells. Nat. Biotechnol..

[37] Blixt O., Head S., Mondala T., Scanlan C., Huflejt M.E., Alvarez R., Bryan M.C., Fazio F., Calarese D., Stevens J. (2004). Printed covalent glycan array for ligand profiling of diverse glycan binding proteins. Proc. Natl. Acad. Sci. USA.

[38] Marchetti R., Perez S., Arda A., Imberty A., Jimenez-Barbero J., Silipo A., Molinaro A. (2016). “Rules of Engagement” of Protein–Glycoconjugate Interactions: A Molecular View Achievable by using NMR Spectroscopy and Molecular Modeling. ChemistryOpen.

[39] Gimeno A., Valverde P., Ardá A., Jiménez-Barbero J. (2020). Glycan structures and their interactions with proteins. A NMR view. Curr. Opin. Struct. Biol..

[40] Mayer M., Meyer B. (2001). Group Epitope Mapping by Saturation Transfer Difference NMR To Identify Segments of a Ligand in Direct Contact with a Protein Receptor. J. Am. Chem. Soc..

[41] Yu Y., Delbianco M. (2020). Conformational Studies of Oligosaccharides. Chem. Eur. J..

[42] Zhang Y., Yamaguchi T., Kato K. (2013). New NMR Tools for Characterizing the Dynamic Conformations and Interactions of Oligosaccharides. Chem. Lett..

[43] Yamaguchi T., Sakae Y., Zhang Y., Yamamoto S., Okamoto Y., Kato K. (2014). Exploration of Conformational Spaces of High-Mannose-Type Oligosaccharides by an NMR-Validated Simulation. Angew. Chem. Int. Ed..

[44] Canales A., Boos I., Perkams L., Karst L., Luber T., Karagiannis T., Domínguez G., Cañada F.J., Pérez-Castells J., Häussinger D. (2017). Breaking the Limits in Analyzing Carbohydrate Recognition by NMR Spectroscopy: Resolving Branch-Selective Interaction of a Tetra-Antennary N-Glycan with Lectins. Angew. Chem. Int. Ed..

[45] Fernández de Toro B., Peng W., Thompson A.J., Domínguez G., Cañada F.J., Pérez-Castells J., Paulson J.C., Jiménez-Barbero J., Canales Á. (2018). Avenues to Characterize the Interactions of Extended N-Glycans with Proteins by NMR Spectroscopy: The Influenza Hemagglutinin Case. Angew. Chem. Int. Ed..

[46] Canales A., Mallagaray A., Pérez-Castells J., Boos I., Unverzagt C., André S., Gabius H.-J., Cañada F.J., Jiménez-Barbero J. (2013). Breaking Pseudo-Symmetry in Multiantennary Complex N-Glycans Using Lanthanide-Binding Tags and NMR Pseudo-Contact Shifts. Angew. Chem. Int. Ed..

[47] Yamamoto S., Yamaguchi T., Erdélyi M., Griesinger C., Kato K. (2011). Paramagnetic Lanthanide Tagging for NMR Conformational Analyses of N-Linked Oligosaccharides. Chem. Eur. J..

[48] Weiss M., Ott D., Karagiannis T., Weishaupt M., Niemietz M., Eller S., Lott M., Martínez-Orts M., Canales Á., Razi N. (2020). Efficient Chemoenzymatic Synthesis of N-Glycans with a β1,4-Galactosylated Bisecting GlcNAc Motif. ChemBioChem.

[49] Brzezicka K., Echeverria B., Serna S., van Diepen A., Hokke C.H., Reichardt N.-C. (2015). Synthesis and Microarray-Assisted Binding Studies of Core Xylose and Fucose Containing N-Glycans. ACS Chem. Biol..

[50] Echeverria B., Serna S., Achilli S., Vivès C., Pham J., Thépaut M., Hokke C.H., Fieschi F., Reichardt N.-C. (2018). Chemoenzymatic Synthesis of N-glycan Positional Isomers and Evidence for Branch Selective Binding by Monoclonal Antibodies and Human C-type Lectin Receptors. ACS Chem. Biol..

[51] Wu Z., Liu Y., Ma C., Li L., Bai J., Byrd-Leotis L., Lasanajak Y., Guo Y., Wen L., Zhu H. (2016). Identification of the binding roles of terminal and internal glycan epitopes using enzymatically synthesized N-glycans containing tandem epitopes. Org. Biomol. Chem..

[52] Pawar S., Hsu L., Narendar Reddy T., Ravinder M., Ren C.-T., Lin Y.-W., Cheng Y.-Y., Lin T.-W., Hsu T.-L., Wang S.-K. (2020). Synthesis of Asymmetric N-Glycans as Common Core Substrates for Structural Diversification through Selective Enzymatic Glycosylation. ACS Chem. Biol..

[53] Wu Z., Liu Y., Li L., Wan X.-F., Zhu H., Guo Y., Wei M., Guan W., Wang P.G. (2017). Decoding glycan protein interactions by a new class of asymmetric N-glycans. Org. Biomol. Chem..

[54] Li L., Guan W., Zhang G., Wu Z., Yu H., Chen X., Wang P.G. (2019). Microarray analyses of closely related glycoforms reveal different accessibilities of glycan determinants on N-glycan branches. Glycobiology.

[55] Ratner D.M., Adams E.W., Su J., O’Keefe B.R., Mrksich M., Seeberger P.H. (2004). Probing Protein–Carbohydrate Interactions with Microarrays of Synthetic Oligosaccharides. ChemBioChem.

[56] Song X., Xia B., Stowell S.R., Lasanajak Y., Smith D.F., Cummings R.D. (2009). Novel Fluorescent Glycan Microarray Strategy Reveals Ligands for Galectins. Chem. Biol..

[57] Song X., Yu H., Chen X., Lasanajak Y., Tappert M.M., Air G.M., Tiwari V.K., Cao H., Chokhawala H.A., Zheng H. (2011). A Sialylated Glycan Microarray Reveals Novel Interactions of Modified Sialic Acids with Proteins and Viruses. J. Biol. Chem..

[58] Huang W., Wang D., Yamada M., Wang L.-X. (2009). Chemoenzymatic Synthesis and Lectin Array Characterization of a Class of N-Glycan Clusters. J. Am. Chem. Soc..

[59] Gao C., Hanes M.S., Byrd-Leotis L.A., Wei M., Jia N., Kardish R.J., McKitrick T.R., Steinhauer D.A., Cummings R.D. (2019). Unique Binding Specificities of Proteins toward Isomeric Asparagine-Linked Glycans. Cell Chem. Biol..

[60] Peng W., de Vries R.P., Grant O.C., Thompson A.J., McBride R., Tsogtbaatar B., Lee P.S., Razi N., Wilson I.A., Woods R.J. (2017). Recent H3N2 Viruses Have Evolved Specificity for Extended, Branched Human-type Receptors, Conferring Potential for Increased Avidity. Cell Host Microbe.

[61] Wu H.-R., Anwar M.T., Fan C.-Y., Low P.Y., Angata T., Lin C.-C. (2019). Expedient assembly of Oligo-LacNAcs by a sugar nucleotide regeneration system: Finding the role of tandem LacNAc and sialic acid position towards siglec binding. Eur. J. Med. Chem..

[62] Iwaki J., Hirabayashi J. (2018). Carbohydrate-Binding Specificity of Human Galectins: An Overview by Frontal Affinity Chromatography. Trends Glycosci. Glycotechnol..

[63] Stevens J., Blixt O., Paulson J.C., Wilson I.A. (2006). Glycan microarray technologies: Tools to survey host specificity of influenza viruses. Nat. Rev. Microbiol..

[64] Neu U., Bauer J., Stehle T. (2011). Viruses and sialic acids: Rules of engagement. Curr. Opin. Struct. Biol..

[65] Day C.J., Semchenko E.A., Korolik V. (2012). Glycoconjugates play a key role in Campylobacter jejuni Infection: Interactions between host and pathogen. Front. Cell. Infect. Microbiol..

[66] Gulati S., Lasanajak Y., Smith D.F., Cummings R.D., Air G.M. (2014). Glycan array analysis of influenza H1N1 binding and release. Cancer Biomark.

[67] Stencel-Baerenwald J.E., Reiss K., Reiter D.M., Stehle T., Dermody T.S. (2014). The sweet spot: Defining virus–sialic acid interactions. Nat. Rev. Microbiol..

[68] Ramani S., Hu L., Venkataram Prasad B.V., Estes M.K. (2016). Diversity in Rotavirus–Host Glycan Interactions: A “Sweet” Spectrum. Cell. Mol. Gastroenterol. Hepatol..

[69] Tzarum N., McBride R., Nycholat C.M., Peng W., Paulson J.C., Wilson I.A. (2017). Unique Structural Features of Influenza Virus H15 Hemagglutinin. J. Virol..

[70] Zhu X., Turner H.L., Lang S., McBride R., Bangaru S., Gilchuk I.M., Yu W., Paulson J.C., Crowe J.E., Ward A.B. (2019). Structural Basis of Protection against H7N9 Influenza Virus by Human Anti-N9 Neuraminidase Antibodies. Cell Host Microbe.

[71] Byrd-Leotis L., Gao C., Jia N., Mehta A.Y., Trost J., Cummings S.F., Heimburg-Molinaro J., Cummings R.D., Steinhauer D.A. (2019). Antigenic Pressure on H3N2 Influenza Virus Drift Strains Imposes Constraints on Binding to Sialylated Receptors but Not Phosphorylated Glycans. J. Virol..

[72] Blixt O., Han S., Liao L., Zeng Y., Hoffmann J., Futakawa S., Paulson J.C. (2008). Sialoside Analogue Arrays for Rapid Identification of High Affinity Siglec Ligands. J. Am. Chem. Soc..

[73] Rillahan C.D., Schwartz E., McBride R., Fokin V.V., Paulson J.C. (2012). Click and Pick: Identification of Sialoside Analogues for Siglec-Based Cell Targeting. Angew. Chem. Int. Ed..

[74] Medve L., Achilli S., Serna S., Zuccotto F., Varga N., Thépaut M., Civera M., Vivès C., Fieschi F., Reichardt N. (2018). On-Chip Screening of a Glycomimetic Library with C-Type Lectins Reveals Structural Features Responsible for Preferential Binding of Dectin-2 over DC-SIGN/R and Langerin. Chemistry.

[75] Ardá A., Blasco P., Varón Silva D., Schubert V., André S., Bruix M., Cañada F.J., Gabius H.-J., Unverzagt C., Jiménez-Barbero J. (2013). Molecular Recognition of Complex-Type Biantennary N-Glycans by Protein Receptors: A Three-Dimensional View on Epitope Selection by NMR. J. Am. Chem. Soc..

[76] Blaum B.S., Hannan J.P., Herbert A.P., Kavanagh D., Uhrín D., Stehle T. (2015). Structural basis for sialic acid–mediated self-recognition by complement factor H. Nat. Chem. Biol..

[77] Macchi E., Rudd T.R., Raman R., Sasisekharan R., Yates E.A., Naggi A., Guerrini M., Elli S. (2016). Nuclear Magnetic Resonance and Molecular Dynamics Simulation of the Interaction between Recognition Protein H7 of the Novel Influenza Virus H7N9 and Glycan Cell Surface Receptors. Biochemistry.

[78] Di Carluccio C., Crisman E., Manabe Y., Forgione R.E., Lacetera A., Amato J., Pagano B., Randazzo A., Zampella A., Lanzetta R. (2020). Characterisation of the Dynamic Interactions between Complex N-Glycans and Human CD22. ChemBioChem.

[79] Forgione R.E., Di Carluccio C., Guzmán-Caldentey J., Gaglione R., Battista F., Chiodo F., Manabe Y., Arciello A., Del Vecchio P., Fukase K. (2020). Unveiling Molecular Recognition of Sialoglycans by Human Siglec-10. iScience.

[80] Gimeno A., Reichardt N.-C., Cañada F.J., Perkams L., Unverzagt C., Jiménez-Barbero J., Ardá A. (2017). NMR and Molecular Recognition of N-Glycans: Remote Modifications of the Saccharide Chain Modulate Binding Features. ACS Chem. Biol..

[81] Diercks T., Infantino A.S., Unione L., Jiménez-Barbero J., Oscarson S., Gabius H.-J. (2018). Fluorinated Carbohydrates as Lectin Ligands: Synthesis of OH/F-Substituted N-Glycan Core Trimannoside and Epitope Mapping by 2D STD-TOCSYreFNMR spectroscopy. Chem. Eur. J..

[82] Manabe Y., Marchetti R., Takakura Y., Nagasaki M., Nihei W., Takebe T., Tanaka K., Kabayama K., Chiodo F., Hanashima S. (2019). The Core Fucose on an IgG Antibody is an Endogenous Ligand of Dectin-1. Angew. Chem. Int. Ed..

[83] Kötzler M.P., Blank S., Bantleon F.I., Wienke M., Spillner E., Meyer B. (2013). Donor Assists Acceptor Binding and Catalysis of Human α1,6-Fucosyltransferase. ACS Chem. Biol..

[84] Claasen B., Axmann M., Meinecke R., Meyer B. (2005). Direct Observation of Ligand Binding to Membrane Proteins in Living Cells by a Saturation Transfer Double Difference (STDD) NMR Spectroscopy Method Shows a Significantly Higher Affinity of Integrin αIIbβ3 in Native Platelets than in Liposomes. J. Am. Chem. Soc..

[85] Xia Y., Zhu Q., Jun K.-Y., Wang J., Gao X. (2010). Clean STD-NMR spectrum for improved detection of ligand-protein interactions at low concentration of protein. Magn. Reson. Chem..

[86] Vogtherr M., Peters T. (2000). Application of NMR Based Binding Assays to Identify Key Hydroxy Groups for Intermolecular Recognition. J. Am. Chem. Soc..

[87] Nagae M., Kanagawa M., Morita-Matsumoto K., Hanashima S., Kizuka Y., Taniguchi N., Yamaguchi Y. (2016). Atomic visualization of a flipped-back conformation of bisected glycans bound to specific lectins. Sci. Rep..

[88] Re S., Miyashita N., Yamaguchi Y., Sugita Y. (2011). Structural Diversity and Changes in Conformational Equilibria of Biantennary Complex-Type N-Glycans in Water Revealed by Replica-Exchange Molecular Dynamics Simulation. Biophys. J..

[89] Nishima W., Miyashita N., Yamaguchi Y., Sugita Y., Re S. (2012). Effect of Bisecting GlcNAc and Core Fucosylation on Conformational Properties of Biantennary Complex-Type N-Glycans in Solution. J. Phys. Chem. B.

[90] Davis B.G. (2002). Synthesis of Glycoproteins. Chem. Rev..

[91] Unverzagt C., Kajihara Y. (2013). Chemical assembly of N-glycoproteins: A refined toolbox to address a ubiquitous posttranslational modification. Chem. Soc. Rev..

[92] Wang L.-X., Amin M.N. (2014). Chemical and Chemoenzymatic Synthesis of Glycoproteins for Deciphering Functions. Chem. Biol..

[93] Carlo U., Yasuhiro K. (2018). Recent advances in the chemical synthesis of N-linked glycoproteins. Curr. Opin. Chem. Biol..

[94] Li Y., Tran A.H., Danishefsky S.J., Tan Z., Shukla A.K. (2019). Chapter Twelve—Chemical biology of glycoproteins: From chemical synthesis to biological impact. Methods Enzymol.

[95] Fairbanks A.J. (2017). The ENGases: Versatile biocatalysts for the production of homogeneous N-linked glycopeptides and glycoproteins. Chem. Soc. Rev..

[96] Takeda Y., Totani K., Matsuo I., Ito Y. (2009). Chemical approaches toward understanding glycan-mediated protein quality control. Curr. Opin. Chem. Biol..

[97] Ito Y., Takeda Y., Seko A., Izumi M., Kajihara Y. (2015). Functional analysis of endoplasmic reticulum glucosyltransferase (UGGT): Synthetic chemistry’s initiative in glycobiology. Semin. Cell Dev. Biol..

[98] Jaeken J., Matthijs G. (2007). Congenital Disorders of Glycosylation: A Rapidly Expanding Disease Family. Annu. Rev. Genomics Hum. Genet..

[99] Mori K. (2015). The unfolded protein response: The dawn of a new field. Proc. Jpn. Acad. Ser. B.

[100] Totani K., Matsuo I., Ito Y. (2004). Tight binding ligand approach to oligosaccharide-grafted protein. Bioorg. Med. Chem. Lett..

[101] Totani K., Matsuo I., Ihara Y., Ito Y. (2006). High-mannose-type glycan modifications of dihydrofolate reductase using glycan–methotrexate conjugates. Biorg. Med. Chem..

[102] Totani K., Ihara Y., Matsuo I., Koshino H., Ito Y. (2005). Synthetic Substrates for an Endoplasmic Reticulum Protein-Folding Sensor, UDP-Glucose: Glycoprotein Glucosyltransferase. Angew. Chem. Int. Ed..

[103] Totani K., Ihara Y., Tsujimoto T., Matsuo I., Ito Y. (2009). The Recognition Motif of the Glycoprotein-Folding Sensor Enzyme UDP-Glc:Glycoprotein Glucosyltransferase. Biochemistry.

[104] Sakono M., Seko A., Takeda Y., Hachisu M., Ito Y. (2012). Biophysical properties of UDP-glucose: Glycoprotein glucosyltransferase, a folding sensor enzyme in the ER, delineated by synthetic probes. Biochem. Biophys. Res. Commun..

[105] Izumi M., Makimura Y., Dedola S., Seko A., Kanamori A., Sakono M., Ito Y., Kajihara Y. (2012). Chemical Synthesis of Intentionally Misfolded Homogeneous Glycoprotein: A Unique Approach for the Study of Glycoprotein Quality Control. J. Am. Chem. Soc..

[106] Izumi M., Kuruma R., Okamoto R., Seko A., Ito Y., Kajihara Y. (2017). Substrate Recognition of Glycoprotein Folding Sensor UGGT Analyzed by Site-Specifically 15N-Labeled Glycopeptide and Small Glycopeptide Library Prepared by Parallel Native Chemical Ligation. J. Am. Chem. Soc..

[107] Kiuchi T., Izumi M., Mukogawa Y., Shimada A., Okamoto R., Seko A., Sakono M., Takeda Y., Ito Y., Kajihara Y. (2018). Monitoring of Glycoprotein Quality Control System with a Series of Chemically Synthesized Homogeneous Native and Misfolded Glycoproteins. J. Am. Chem. Soc..

[108] Huang C., Harada Y., Hosomi A., Masahara-Negishi Y., Seino J., Fujihira H., Funakoshi Y., Suzuki T., Dohmae N., Suzuki T. (2015). Endo-β-N-acetylglucosaminidase forms N-GlcNAc protein aggregates during ER-associated degradation in Ngly1-defective cells. Proc. Natl. Acad. Sci. USA.

[109] Maynard J.C., Fujihira H., Dolgonos G.E., Suzuki T., Burlingame A.L. (2020). Cytosolic N-GlcNAc proteins are formed by the action of endo-β-N-acetylglucosaminidase. Biochem. Biophys. Res. Commun..

[110] Bi Y., Might M., Vankayalapati H., Kuberan B. (2017). Repurposing of Proton Pump Inhibitors as first identified small molecule inhibitors of endo-β-N-acetylglucosaminidase (ENGase) for the treatment of NGLY1 deficiency, a rare genetic disease. Bioorg. Med. Chem. Lett..

[111] Fujihira H., Masahara-Negishi Y., Tamura M., Huang C., Harada Y., Wakana S., Takakura D., Kawasaki N., Taniguchi N., Kondoh G. (2017). Lethality of mice bearing a knockout of the Ngly1-gene is partially rescued by the additional deletion of the Engase gene. PLoS Genet..

[112] Wang P., Dong S., Shieh J.-H., Peguero E., Hendrickson R., Moore M.A.S., Danishefsky S.J. (2013). Erythropoietin Derived by Chemical Synthesis. Science.

[113] Okamoto R., Mandal K., Ling M., Luster A.D., Kajihara Y., Kent S.B.H. (2014). Total Chemical Synthesis and Biological Activities of Glycosylated and Non-Glycosylated Forms of the Chemokines CCL1 and Ser-CCL1. Angew. Chem. Int. Ed..

[114] Reif A., Siebenhaar S., Tröster A., Schmälzlein M., Lechner C., Velisetty P., Gottwald K., Pöhner C., Boos I., Schubert V. (2014). Semisynthesis of Biologically Active Glycoforms of the Human Cytokine Interleukin 6. Angew. Chem. Int. Ed..

[115] Murakami M., Kiuchi T., Nishihara M., Tezuka K., Okamoto R., Izumi M., Kajihara Y. (2016). Chemical synthesis of erythropoietin glycoforms for insights into the relationship between glycosylation pattern and bioactivity. Sci. Adv..

[116] Streichert K., Seitz C., Hoffmann E., Boos I., Jelkmann W., Brunner T., Unverzagt C., Rubini M. (2019). Synthesis of Erythropoietins Site-Specifically Conjugated with Complex-Type N-Glycans. ChemBioChem.

[117] Sakamoto I., Tezuka K., Fukae K., Ishii K., Taduru K., Maeda M., Ouchi M., Yoshida K., Nambu Y., Igarashi J. (2012). Chemical Synthesis of Homogeneous Human Glycosyl-interferon-β That Exhibits Potent Antitumor Activity In Vivo. J. Am. Chem. Soc..

[118] Murakami M., Okamoto R., Izumi M., Kajihara Y. (2012). Chemical Synthesis of an Erythropoietin Glycoform Containing a Complex-type Disialyloligosaccharide. Angew. Chem. Int. Ed..

[119] Yang Q., An Y., Zhu S., Zhang R., Loke C.M., Cipollo J.F., Wang L.-X. (2017). Glycan Remodeling of Human Erythropoietin (EPO) Through Combined Mammalian Cell Engineering and Chemoenzymatic Transglycosylation. ACS Chem. Biol..

[120] Maki Y., Okamoto R., Izumi M., Kajihara Y. (2020). Chemical Synthesis of an Erythropoietin Glycoform Having a Triantennary N-Glycan: Significant Change of Biological Activity of Glycoprotein by Addition of a Small Molecular Weight Trisaccharide. J. Am. Chem. Soc..

[121] Macmillan D., Bill R.M., Sage K.A., Fern D., Flitsch S.L. (2001). Selective in vitro glycosylation of recombinant proteins: Semi-synthesis of novel homogeneous glycoforms of human erythropoietin. Chem. Biol..

[122] Kochendoerfer G.G., Chen S.-Y., Mao F., Cressman S., Traviglia S., Shao H., Hunter C.L., Low D.W., Cagle E.N., Carnevali M. (2003). Design and Chemical Synthesis of a Homogeneous Polymer-Modified Erythropoiesis Protein. Science.

[123] Hirano K., Macmillan D., Tezuka K., Tsuji T., Kajihara Y. (2009). Design and Synthesis of a Homogeneous Erythropoietin Analogue with Two Human Complex-Type Sialyloligosaccharides: Combined Use of Chemical and Bacterial Protein Expression Methods. Angew. Chem. Int. Ed..

[124] Lee D.J., Cameron A.J., Wright T.H., Harris P.W.R., Brimble M.A. (2019). A synthetic approach to ‘click’ neoglycoprotein analogues of EPO employing one-pot native chemical ligation and CuAAC chemistry. Chem. Sci..

[125] Tanaka K., Siwu E.R.O., Minami K., Hasegawa K., Nozaki S., Kanayama Y., Koyama K., Chen W.C., Paulson J.C., Watanabe Y. (2010). Noninvasive Imaging of Dendrimer-Type N-Glycan Clusters: In Vivo Dynamics Dependence on Oligosaccharide Structure. Angew. Chem. Int. Ed..

[126] Vong K., Yamamoto T., Tanaka K. (2020). Artificial Glycoproteins as a Scaffold for Targeted Drug Therapy. Small.

[127] Valverde P., Ardá A., Reichardt N.-C., Jiménez-Barbero J., Gimeno A. (2019). Glycans in drug discovery. MedChemComm.

[128] Eon-Duval A., Broly H., Gleixner R. (2012). Quality attributes of recombinant therapeutic proteins: An assessment of impact on safety and efficacy as part of a quality by design development approach. Biotechnol. Progr..

[129] Beck A., Wagner-Rousset E., Ayoub D., Van Dorsselaer A., Sanglier-Cianférani S. (2013). Characterization of Therapeutic Antibodies and Related Products. Anal. Chem..

[130] Shinkawa T., Nakamura K., Yamane N., Shoji-Hosaka E., Kanda Y., Sakurada M., Uchida K., Anazawa H., Satoh M., Yamasaki M. (2003). The Absence of Fucose but Not the Presence of Galactose or Bisecting N-Acetylglucosamine of Human IgG1 Complex-type Oligosaccharides Shows the Critical Role of Enhancing Antibody-dependent Cellular Cytotoxicity. J. Biol. Chem..

[131] Shields R.L., Lai J., Keck R., O’Connell L.Y., Hong K., Meng Y.G., Weikert S.H.A., Presta L.G. (2002). Lack of Fucose on Human IgG1 N-Linked Oligosaccharide Improves Binding to Human FcγRIII and Antibody-dependent Cellular Toxicity. J. Biol. Chem..

[132] Satoh M., Shitara K., Hanai N. (2006). The Current Stream and Prospect of Glycoscience Application Therapeutic Antibodies. Trends Glycosci. Glycotechnol..

[133] Niwa R., Shoji-Hosaka E., Sakurada M., Shinkawa T., Uchida K., Nakamura K., Matsushima K., Ueda R., Hanai N., Shitara K. (2004). Defucosylated Chimeric Anti-CC Chemokine Receptor 4 IgG1 with Enhanced Antibody-Dependent Cellular Cytotoxicity Shows Potent Therapeutic Activity to T-Cell Leukemia and Lymphoma. Cancer Res..

[134] Davies J., Jiang L., Pan L.-Z., LaBarre M.J., Anderson D., Reff M. (2001). Expression of GnTIII in a recombinant anti-CD20 CHO production cell line: Expression of antibodies with altered glycoforms leads to an increase in ADCC through higher affinity for FCγRIII. Biotechnol. Bioeng..

[135] Hodoniczky J., Zheng Y.Z., James D.C. (2005). Control of Recombinant Monoclonal Antibody Effector Functions by Fc N-Glycan Remodeling *In Vitro*. Biotechnol. Progr..

[136] Ferrara C., Brünker P., Suter T., Moser S., Püntener U., Umaña P. (2006). Modulation of therapeutic antibody effector functions by glycosylation engineering: Influence of Golgi enzyme localization domain and co-expression of heterologous β1, 4-N-acetylglucosaminyltransferase III and Golgi α-mannosidase II. Biotechnol. Bioeng..

[137] Huang W., Giddens J., Fan S.-Q., Toonstra C., Wang L.-X. (2012). Chemoenzymatic Glycoengineering of Intact IgG Antibodies for Gain of Functions. J. Am. Chem. Soc..

[138] Qasba P.K. (2015). Glycans of Antibodies as a Specific Site for Drug Conjugation Using Glycosyltransferases. Bioconjugate Chem..

[139] Manabe S. (2020). Attempts to synthesize homogeneous glycan-conjugated antibody-drug conjugates. Transl. Regul. Sci..

[140] Wang L.-X., Tong X., Li C., Giddens J.P., Li T. (2019). Glycoengineering of Antibodies for Modulating Functions. Annu. Rev. Biochem.

[141] Manabe S., Yamaguchi Y., Matsumoto K., Fuchigami H., Kawase T., Hirose K., Mitani A., Sumiyoshi W., Kinoshita T., Abe J. (2019). Characterization of Antibody Products Obtained through Enzymatic and Nonenzymatic Glycosylation Reactions with a Glycan Oxazoline and Preparation of a Homogeneous Antibody–Drug Conjugate via Fc N-Glycan. Bioconjugate Chem..

[142] Parsons T.B., Struwe W.B., Gault J., Yamamoto K., Taylor T.A., Raj R., Wals K., Mohammed S., Robinson C.V., Benesch J.L.P. (2016). Optimal Synthetic Glycosylation of a Therapeutic Antibody. Angew. Chem. Int. Ed..

[143] Boeggeman E., Ramakrishnan B., Pasek M., Manzoni M., Puri A., Loomis K.H., Waybright T.J., Qasba P.K. (2009). Site Specific Conjugation of Fluoroprobes to the Remodeled Fc N-Glycans of Monoclonal Antibodies Using Mutant Glycosyltransferases: Application for Cell Surface Antigen Detection. Bioconjugate Chem..

[144] Tang F., Yang Y., Tang Y., Tang S., Yang L., Sun B., Jiang B., Dong J., Liu H., Huang M. (2016). One-pot N-glycosylation remodeling of IgG with non-natural sialylglycopeptides enables glycosite-specific and dual-payload antibody–drug conjugates. Org. Biomol. Chem..

[145] Zeglis B.M., Davis C.B., Aggeler R., Kang H.C., Chen A., Agnew B.J., Lewis J.S. (2013). Enzyme-Mediated Methodology for the Site-Specific Radiolabeling of Antibodies Based on Catalyst-Free Click Chemistry. Bioconjugate Chem..

[146] van Geel R., Wijdeven M.A., Heesbeen R., Verkade J.M.M., Wasiel A.A., van Berkel S.S., van Delft F.L. (2015). Chemoenzymatic Conjugation of Toxic Payloads to the Globally Conserved N-Glycan of Native mAbs Provides Homogeneous and Highly Efficacious Antibody–Drug Conjugates. Bioconjugate Chem..

[147] Lo H.-J., Krasnova L., Dey S., Cheng T., Liu H., Tsai T.-I., Wu K.B., Wu C.-Y., Wong C.-H. (2019). Synthesis of Sialidase-Resistant Oligosaccharide and Antibody Glycoform Containing α2,6-Linked 3Fax-Neu5Ac. J. Am. Chem. Soc..

[148] Hossain M.A., Okamoto R., Karas J.A., Praveen P., Liu M., Forbes B.E., Wade J.D., Kajihara Y. (2020). Total Chemical Synthesis of a Nonfibrillating Human Glycoinsulin. J. Am. Chem. Soc..

[149] Wang L.-X., Ni J., Singh S., Li H. (2004). Binding of High-Mannose-Type Oligosaccharides and Synthetic Oligomannose Clusters to Human Antibody 2G12: Implications for HIV-1 Vaccine Design. Chem. Biol..

[150] Amin M.N., McLellan J.S., Huang W., Orwenyo J., Burton D.R., Koff W.C., Kwong P.D., Wang L.-X. (2013). Synthetic glycopeptides reveal the glycan specificity of HIV-neutralizing antibodies. Nat. Chem. Biol..

[151] Aussedat B., Vohra Y., Park P.K., Fernández-Tejada A., Alam S.M., Dennison S.M., Jaeger F.H., Anasti K., Stewart S., Blinn J.H. (2013). Chemical Synthesis of Highly Congested gp120 V1V2 N-Glycopeptide Antigens for Potential HIV-1-Directed Vaccines. J. Am. Chem. Soc..

[152] Cai H., Orwenyo J., Giddens J.P., Yang Q., Zhang R., LaBranche C.C., Montefiori D.C., Wang L.-X. (2017). Synthetic Three-Component HIV-1 V3 Glycopeptide Immunogens Induce Glycan-Dependent Antibody Responses. Cell Chem. Biol..

[153] Cai H., Zhang R.-S., Orwenyo J., Giddens J., Yang Q., LaBranche C.C., Montefiori D.C., Wang L.-X. (2018). Synthetic HIV V3 Glycopeptide Immunogen Carrying a N334 N-Glycan Induces Glycan-Dependent Antibodies with Promiscuous Site Recognition. J. Med. Chem..

[154] Wang C.-C., Chen J.-R., Tseng Y.-C., Hsu C.-H., Hung Y.-F., Chen S.-W., Chen C.-M., Khoo K.-H., Cheng T.-J., Cheng Y.-S.E. (2009). Glycans on influenza hemagglutinin affect receptor binding and immune response. Proc. Natl. Acad. Sci. USA.

[155] Chen J.-R., Yu Y.-H., Tseng Y.-C., Chiang W.-L., Chiang M.-F., Ko Y.-A., Chiu Y.-K., Ma H.-H., Wu C.-Y., Jan J.-T. (2014). Vaccination of monoglycosylated hemagglutinin induces cross-strain protection against influenza virus infections. Proc. Natl. Acad. Sci. USA.

[156] Watanabe Y., Allen J.D., Wrapp D., McLellan J.S., Crispin M. (2020). Site-specific glycan analysis of the SARS-CoV-2 spike. Science.

[157] Brun J., Vasiljevic S., Gangadharan B., Hensen M., Chandran A.V., Hill M.L., Kiappes J.L., Dwek R.A., Alonzi D.S., Struwe W.B. (2020). Analysis of SARS-CoV-2 spike glycosylation reveals shedding of a vaccine candidate. bioRxiv.

[158] Yamazaki N., Kojima S., Bovin N.V., André S., Gabius S., Gabius H.J. (2000). Endogenous lectins as targets for drug delivery. Adv. Drug Del. Rev..

[159] Zhang H., Ma Y., Sun X.-L. (2010). Recent developments in carbohydrate-decorated targeted drug/gene delivery. Med. Res. Revs..

[160] Cecioni S., Imberty A., Vidal S. (2015). Glycomimetics versus Multivalent Glycoconjugates for the Design of High Affinity Lectin Ligands. Chem. Rev..

[161] Ogura A., Tahara T., Nozaki S., Morimoto K., Kizuka Y., Kitazume S., Hara M., Kojima S., Onoe H., Kurbangalieva A. (2016). Visualizing Trimming Dependence of Biodistribution and Kinetics with Homo- and Heterogeneous N-Glycoclusters on Fluorescent Albumin. Sci. Rep..

[162] Tsubokura K., Vong K.K.H., Pradipta A.R., Ogura A., Urano S., Tahara T., Nozaki S., Onoe H., Nakao Y., Sibgatullina R. (2017). In Vivo Gold Complex Catalysis within Live Mice. Angew. Chem. Int. Ed..

[163] Eda S., Nasibullin I., Vong K., Kudo N., Yoshida M., Kurbangalieva A., Tanaka K. (2019). Biocompatibility and therapeutic potential of glycosylated albumin artificial metalloenzymes. Nat. Catal..

[164] Chen W.C., Completo G.C., Sigal D.S., Crocker P.R., Saven A., Paulson J.C. (2010). In Vivo targeting of B-cell lymphoma with glycan ligands of CD22. Blood.

[165] Macauley M.S., Pfrengle F., Rademacher C., Nycholat C.M., Gale A.J., von Drygalski A., Paulson J.C. (2013). Antigenic liposomes displaying CD22 ligands induce antigen-specific B cell apoptosis. J. Clin. Investig..

[166] Kawasaki N., Rillahan C.D., Cheng T.-Y., Van Rhijn I., Macauley M.S., Moody D.B., Paulson J.C. (2014). Targeted Delivery of Mycobacterial Antigens to Human Dendritic Cells via Siglec-7 Induces Robust T Cell Activation. J. Immunol..

[167] Angata T., Nycholat C.M., Macauley M.S. (2015). Therapeutic Targeting of Siglecs using Antibody- and Glycan-Based Approaches. Trends Pharmacol. Sci..

[168] Peng W., Paulson J.C. (2017). CD22 Ligands on a Natural N-Glycan Scaffold Efficiently Deliver Toxins to B-Lymphoma Cells. J. Am. Chem. Soc..

[169] Nycholat C.M., Duan S., Knuplez E., Worth C., Elich M., Yao A., O’Sullivan J., McBride R., Wei Y., Fernandes S.M. (2019). A Sulfonamide Sialoside Analogue for Targeting Siglec-8 and -F on Immune Cells. J. Am. Chem. Soc..

[170] Wang X., Lang S., Tian Y., Zhang J., Yan X., Fang Z., Weng J., Lu N., Wu X., Li T. (2020). Glycoengineering of Natural Killer Cells with CD22 Ligands for Enhanced Anticancer Immunotherapy. ACS Cent. Sci..

[171] Wang H., Huang W., Orwenyo J., Banerjee A., Vasta G.R., Wang L.-X. (2013). Design and synthesis of glycoprotein-based multivalent glyco-ligands for influenza hemagglutinin and human galectin-3. Biorg. Med. Chem..

[172] Cagnoni A.J., Pérez Sáez J.M., Rabinovich G.A., Mariño K.V. (2016). Turning-Off Signaling by Siglecs, Selectins, and Galectins: Chemical Inhibition of Glycan-Dependent Interactions in Cancer. Front. Oncol..

[173] Laaf D., Bojarová P., Elling L., Křen V. (2019). Galectin–Carbohydrate Interactions in Biomedicine and Biotechnology. Trends Biotechnol..

[174] Griffin M.E., Hsieh-Wilson L.C. (2016). Glycan Engineering for Cell and Developmental Biology. Cell Chem. Biol..

[175] Prescher J.A., Dube D.H., Bertozzi C.R. (2004). Chemical remodelling of cell surfaces in living animals. Nature.

[176] Wang H., Mooney D.J. (2020). Metabolic glycan labelling for cancer-targeted therapy. Nat. Chem..

[177] Zeng Y., Ramya T.N.C., Dirksen A., Dawson P.E., Paulson J.C. (2009). High-efficiency labeling of sialylated glycoproteins on living cells. Nat. Methods.

[178] Hui J., Bao L., Li S., Zhang Y., Feng Y., Ding L., Ju H. (2017). Localized Chemical Remodeling for Live Cell Imaging of Protein-Specific Glycoform. Angew. Chem. Int. Ed..

[179] Boyce M., Carrico I.S., Ganguli A.S., Yu S.-H., Hangauer M.J., Hubbard S.C., Kohler J.J., Bertozzi C.R. (2011). Metabolic cross-talk allows labeling of O-linked β-N-acetylglucosamine-modified proteins via the N-acetylgalactosamine salvage pathway. Proc. Natl. Acad. Sci. USA.

[180] Zheng T., Jiang H., Gros M., Soriano del Amo D., Sundaram S., Lauvau G., Marlow F., Liu Y., Stanley P., Wu P. (2011). Tracking N-Acetyllactosamine on Cell-Surface Glycans In Vivo. Angew. Chem. Int. Ed..

[181] Mbua N.E., Li X., Flanagan-Steet H.R., Meng L., Aoki K., Moremen K.W., Wolfert M.A., Steet R., Boons G.-J. (2013). Selective Exo-Enzymatic Labeling of N-Glycans on the Surface of Living Cells by Recombinant ST6Gal I. Angew. Chem. Int. Ed..

[182] Briard J.G., Jiang H., Moremen K.W., Macauley M.S., Wu P. (2018). Cell-based glycan arrays for probing glycan–glycan binding protein interactions. Nat. Commun..

[183] Tang F., Zhou M., Qin K., Shi W., Yashinov A., Yang Y., Yang L., Guan D., Zhao L., Tang Y. (2020). Selective N-glycan editing on living cell surfaces to probe glycoconjugate function. Nat. Chem. Biol..

[184] Frame T., Carroll T., Korchagina E., Bovin N., Henry S. (2007). Synthetic glycolipid modification of red blood cell membranes. Transfusion.

[185] Huang M.L., Smith R.A.A., Trieger G.W., Godula K. (2014). Glycocalyx Remodeling with Proteoglycan Mimetics Promotes Neural Specification in Embryonic Stem Cells. J. Am. Chem. Soc..

[186] Hudak J.E., Canham S.M., Bertozzi C.R. (2014). Glycocalyx engineering reveals a Siglec-based mechanism for NK cell immunoevasion. Nat. Chem. Biol..

[187] Paszek M.J., DuFort C.C., Rossier O., Bainer R., Mouw J.K., Godula K., Hudak J.E., Lakins J.N., Wijekoon A.C., Cassereau L. (2014). The cancer glycocalyx mechanically primes integrin-mediated growth and survival. Nature.

[188] Pulsipher A., Griffin M.E., Stone S.E., Brown J.M., Hsieh-Wilson L.C. (2014). Directing Neuronal Signaling through Cell-Surface Glycan Engineering. J. Am. Chem. Soc..

[189] Pulsipher A., Griffin M.E., Stone S.E., Hsieh-Wilson L.C. (2015). Long-Lived Engineering of Glycans to Direct Stem Cell Fate. Angew. Chem. Int. Ed..

[190] Latypova L., Sibgatullina R., Ogura A., Fujiki K., Khabibrakhmanova A., Tahara T., Nozaki S., Urano S., Tsubokura K., Onoe H. (2017). Sequential Double “Clicks” toward Structurally Well-Defined Heterogeneous N-Glycoclusters: The Importance of Cluster Heterogeneity on Pattern Recognition In Vivo. Adv. Sci..

